# Palstimolide A: A Complex Polyhydroxy Macrolide with Antiparasitic Activity

**DOI:** 10.3390/molecules25071604

**Published:** 2020-03-31

**Authors:** Lena Keller, Jair L. Siqueira-Neto, Julia M. Souza, Korina Eribez, Gregory M. LaMonte, Jennifer E. Smith, William H. Gerwick

**Affiliations:** 1 Center for Marine Biotechnology and Biomedicine, Scripps Institution of Oceanography, University of California San Diego, La Jolla, CA 92037, USA; Lena.Keller@helmholtz-hips.de; 2 Skaggs School of Pharmacy and Pharmaceutical Sciences, University of California San Diego, La Jolla, CA 92093, USA; jairlage@ucsd.edu (J.L.S.-N.); jmsouza@ucsd.edu (J.M.S.); 3 Núcleo de Pesquisas em Ciências Exatas e Tecnológicas, Universidade de Franca, Franca, SP 14404-600, Brazil; greg.lamonte@gmail.com; 4 Department of Pediatrics, School of Medicine, University of California San Diego, La Jolla, CA, 92093, USA; korina.hernandez@yahoo.com; 5 Marine Biology Division, Scripps Institution of Oceanography, University of California San Diego, La Jolla, CA 92037, USA; jes013@ucsd.edu

**Keywords:** natural products, cyanobacteria, antimalarial agents, malaria, leishmaniosis, macrolide

## Abstract

Marine Cyanobacteria (blue-green algae) have been shown to possess an enormous potential to produce structurally diverse natural products that exhibit a broad spectrum of potent biological activities, including cytotoxic, antifungal, antiparasitic, antiviral, and antibacterial activities. Here, we report the isolation and structure determination of palstimolide A, a complex polyhydroxy macrolide with a 40-membered ring that was isolated from a tropical marine cyanobacterium collected at Palmyra Atoll. NMR-guided fractionation in combination with MS^2^-based molecular networking and isolation via HPLC yielded 0.7 mg of the pure compound. The small quantity isolated along with the presence of significant signal degeneracy in both the ^1^H and ^13^C-NMR spectra complicated the structure elucidation of palstimolide A. Various NMR experiments and solvent systems were employed, including the LR-HSQMBC experiment that allows the detection of long-range ^1^H–^13^C correlation data across 4-, 5-, and even 6-bonds. This expanded NMR data set enabled the elucidation of the palstimolide’s planar structure, which is characterized by several 1,5-disposed hydroxy groups as well as a *tert*-butyl group. The compound showed potent antimalarial activity with an IC_50_ of 223 nM as well as interesting anti-leishmanial activity with an IC_50_ of 4.67 µM.

## 1. Introduction

Marine cyanobacteria are extraordinarily rich in biologically active and structurally diverse natural products (NPs), often times deriving from hybrid nonribosomal peptide synthetase (NRPS) and polyketide synthase (PKS) biosynthetic pathways [1,2,3]. However, those cyanobacterial NPs with an exclusive PKS origin are often cyclized to form macrolides, a class of compounds that generally possesses important biomedical properties [4,5]. From other microbial sources, macrolide NPs have proven utility in treating human diseases, such as erythromycin and clarithromycin, as antibiotics, tacrolimus as a macrolide immunosuppressant, and amphotericin B and nystatin as antifungals. Cyanobacteria are also known to produce a variety of structurally diverse macrolides that possess biological activities with potential applications in medicine. These carbon-chain compounds show a high diversity in their skeleton architectures along with complex spatial configurations. Examples of macrolides from Cyanobacteria include bastimolide A and B [6,7], amantelides A and B [8], nuiapolide [9], caylobolide A and B [10,11], swinholide A [12], phormidolide [13], cyanolide A [14], and palmyrolide [15].

Here, we report the newest member of the macrolide class from the marine cyanobacterium *Leptolyngbya* sp., given the trivial name palstimolide A (**1**), a name which reflects its collection from Palmyra Atoll in the Central Pacific and structural relationship to the bastimolides [6,7]. As distinctive structural features, palstimolide A embraces seven 1,5-diol equivalents and one 1,7 diol around a 40-membered macrolide ring, along with a *t*-butyl group at its distal terminus. In vitro, palstimolide A displays potent antimalarial and modest anti-leishmanial activity.

## 2. Results and Discussion

### 2.1. Isolation and Structure Elucidation

Cyanobacterial colonies of the genus *Leptolyngbya* sp. were collected at Palmyra Atoll by shallow water snorkeling. The crude extract was initially fractionated using vacuum liquid chromatography (VLC) as well as solid phase extraction (SPE) for further analysis by MS and NMR. Our discovery strategy to locate natural products with novel structural frameworks included MS^2^-based metabolomics (Molecular Networking) [16] for strain selection and dereplication as well as NMR-guided fractionation for isolation driven by structural features. This approach indicated the presence of an unusual macrolide in the extract of a cyanobacterial field collection from the Palmyra Atoll; HPLC isolation ultimately yielded 0.7 mg of the pure compound. The small quantity isolated along with significant overlap in both the proton and carbon spectra made the NMR-based structure elucidation of **1** quite challenging.

**Figure 1:**
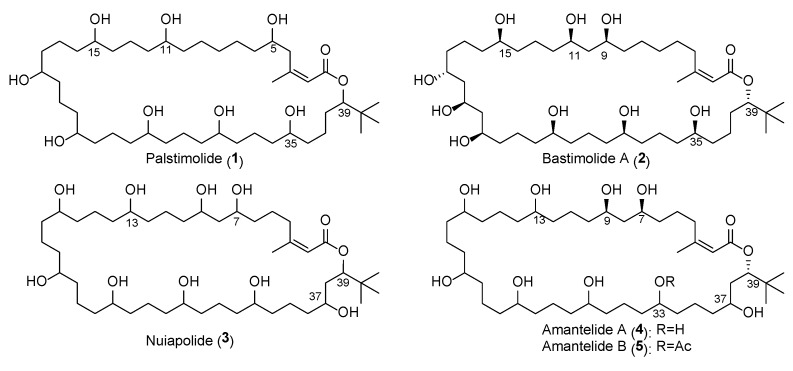
Structure of palstimolide A (**1**) together with the related cyanobacterial 40-membered polyhydroxy macrolides bastimolide A [6], amantelides A and B [8], and nuiapolide [9].

Various NMR experiments and solvent systems were employed to solve the structure of **1**, including the LR-HSQMBC that allows the detection of 4-, 5-, and even 6-bond long-range ^n^J_CH_ heteronuclear couplings [17] as well as 1D TOCSY with different mixing times. Using this expanded NMR data set, palstimolide A (**1**) was characterized as possessing seven contiguous 1,5-diols (or diol equivalents) and a *tert*-butyl substituent (Figure 1), and belonging to a small group of cyanobacterial polyhydroxy macrolides that includes bastimolide A (**2**) and B [6,7], nuiapolide (**3**) [9], and amantelides A (**4**) and B (**5**) [8]. All of these macrolides share a 40-membered ring with several hydroxy groups, a *tert*-butyl moiety, and an α,β–unsaturated carboxyl group (Figure 1). Interestingly, all members of this structural class differ in the arrangement and number of the hydroxy groups with palstimolide A being the only member that contains only 1,5- and 1,7-diols but no 1,3-diol relationships.

HRESIMS of palstimolide A (**1**) displayed an ion peak at *m/z* 773.6182 (calcd for C_44_H_85_O_10_^+^, 773.6137), consistent with the molecular formula C_44_H_84_O_10_ that inherently contains three double-bond equivalents (DBE). The ^1^H-NMR spectrum of **1** in pyridine-*d_5_* (Figure S1, Supplementary Materials) exhibited highly overlapping signals characteristic of a polyhydroxy macrolide, including broad signals indicative of eight hydroxy groups at δ 5.82 (1H), 5.74 (6H), and 5.66 (1H) along with oxymethine signals at 4.21 (1H, broad), 3.93-4.00 (5H, overlap), and 3.87 (2H, overlap). Another downfield shifted oxymethine signal at δ5.08 (1H, dd, *J* = 10.3, 1.7 Hz) indicated an ester bond and a downfield singlet at δ6.01 (1H, s) indicated the presence of a conjugated double bond. The proton NMR spectrum also showed several overlapping methylene signals between 1.4 and 1.9 ppm, an olefinic methyl signal at δ2.09 (3H, s) as well as a large singlet at δ0.94 (9H, s) indicative of a *tert*-butyl group. The HSQC spectrum revealed the presence of a downfield shifted methylene group (δ_C-4_ 42.0, δ_H-4_ 3.38/2.92) in addition to six methylene groups (δ_C-7_ 23.5, δ_H-7_ 1.80/1.58, δ_C-8_ 30.4, δ_H-8_ 1.48/1.42, δ_C-9_ 26.5, δ_H-9_ 1.74/1.58, δ_C-10_ 38.4, δ_H-10_ 1.69, δ_C-37_ 23.5, δ_H-37_ 1.92/1.60, δ_C-39_ 30.2, δ_H-39_ 1.70/1.64) and two highly-crowded methylene areas (δ_C_ 38.7–38.2, δ_H_ 1.82–1.75, δ_C_ 22.8–22.4, δ_H_ 2.15–2.04/1.89–1.76).

In-depth analysis of the standard 2D-NMR data together with more specific NMR experiments, such as the LR-HSQMBC [17] and utilization of different solvent systems, allowed elucidation of the planar structure of palstimolide A (**1**). A sequential spin system starting from the methylene protons at δ3.38/2.92 (H-4) and followed by an oxymethine proton at δ4.21 (H-5), methylene protons at δ1.78 (H-6), and two methylene protons at δ1.80/1.58 (H-7), were deduced from TOCSY and COSY spectra. Together with HMBC signals, the spin system was extended with three more methylene groups to another oxymethine proton at δ3.87 (H-11), allowing the assignment of the 1,7-diol unit. HMBC correlations from the methine proton at δ6.01 (H-2) to a carbonyl carbon at δ167.1 (C-1), a quarternary carbon at δ159.9 (C-3), the methylene group at δ42.0 (C-4), and a methyl group at δ26.7 (Me-44) revealed an α,β-unsaturated carbonyl functionality connected to the 1,7-diol unit.

A 1,5-diol moiety was assembled using successive TOCSY and COSY correlations from H35 to H39. Due to overlapping signals in the methylene region, an LR-HSQMBC experiment [17] was employed (Figure S6, Supplementary Materials) to confirm the 1,5-diol moiety showing a long-range ^5^J_CH_ heteronuclear coupling from the proton at δ5.09 (H-39) to the oxymethine carbon at δ71.1 (H-35). HMBC correlations from H39 to another quaternary carbon at δ34.6 as well as to three isochronous methyl groups at δ26.0 allowed the extension of the structure to include the *tert*-butyl moiety. Moreover, an HMBC correlation from H39 to the carbonyl carbon at δ167.1 (C-1) linked these partial structures via an ester bond.

**Figure 2:**
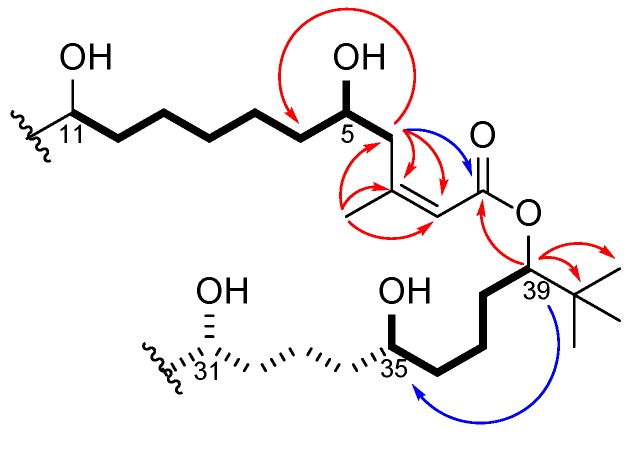
Key correlations deduced from COSY/TOCSY (bolded bonds), HMBC data (red arrows), and LR-HSQMBC data (blue arrows) leading to the partial structure of palstimolide A (**1**). The dashed bond section was assembled from 1D TOCSY data in MeOH-*d*_4_, which connected H35 with H31.

In parallel to the 2D COSY and TOCSY experiments, a set of 1D-TOCSY experiments, with selective irradiation of H5, H35, and H39 with different mixing times between 20 and 150 ms (Figures S9–S11, Supplementary Materials), were recorded in methanol-*d4* and helped assign the spin systems involving each of the individual oxymethines. These spectra also included information concerning the distance between the resonances; as the mixing times lengthened the correlations with more distant protons were observed [18]. Methanol-*d4* was used as a solvent because the proton signals for H5 (δ4.21) and H35 (δ4.21) were separated from the other oxymethine signals (δ3.56–3.54) in this solvent. This data set confirmed the locations of the 1,7-diol and 1,5-diol units as shown with bolded bonds in Figure 2.

The substructure shown in Figure 2 accounted for C_31_H_64_O_6_, leaving a C_13_H_20_O_4_ portion of the molecular formula to be deduced as follows. Three sets of almost identical chemical shifts belonging to chemically equivalent structural units were unassigned at this point: one set of methylene signals (G_1_: δ_C_ 22.8–22.4, δ_H_ 2.15–2.04/1.89–1.76), another set of downfield shifted methylene signals (G_2_: δ_C_ 38.7–38.2, δ_H_ 1.82–1.73), and a set of signals accounting for five oxymethine groups (G_3_: δ_C_ 71.2–70.8, δ_H_ 4.00–3.93) (this latter band includes C-31, as pictured in Figure 2, as well as four additional oxymethines). In addition, five of the six protons that accounted for the OH signal at δ_H_ 5.74 remained unassigned. HMBC correlations between all three groups of protons as well as COSY correlations between G_1_ and G_2_, between G_2_ and G_3_, and between G_3_ and the hydroxy signal at δ_H_ 5.74 allowed assignment of another four 1-5-diol groups. In addition, MS/MS fragmentation showed the loss of eight 18 mass unit fragments, indicating the presence of a total of eight free hydroxy groups about the macrolide ring. Considering the molecular formula and the remaining NMR data, 1,5-diol relationships were required to connect the partial structure in Figure 2 to a 40-membered polyhydroxy macrolide, as shown in Figure 1. Unfortunately, the absolute configuration of palstimolide (**1**) could not be determined due to the small amount isolated and its complex molecular structure.

### 2.2. Bioactivity Testing

Palstimolide A (**1**) showed potent antimalarial activity against the blood stage of the malaria parasite *Plasmodium falciparum* Dd2 (IC_50_ = 172.5 nM) in combination with a low toxicity to liver HepG2 cells (IC_50_ = 5040 nM), resulting in a selectivity index of 29.2. The macrolide also showed activity against the intracellular *Leishmania donovani* parasite infecting murine macrophage cells (IC_50_ = 4.67 μM) with at least a 2-fold toxicity window (the cytotoxic concentration 50% (CC_50_) was above the maximum tested concentration of 10 µM) (Table 1). Even though the selectivity towards the host cells was only moderate, it is remarkable that palstimolide A resulted in more than 95% intracellular elimination at 5 µM in vitro. This indicates that the compound was able to eliminate 95% of the parasites compared to untreated control cells (=0% efficacy) and non-infected controls (=100% efficacy). Parasite reduction is an important measurement to predict the in vivo efficacy of a compound. In the future, we hope to conduct a pharmacokinetic analysis on the compound followed by an in vivo antiparasitic efficacy model using infected mice; however, this requires the availability of additional compound supplies, perhaps through total chemical synthesis. The related metabolite, bastimolide A (**2**), was previously shown to exhibit a very similar biological profile compared to palstimolide A against several *Plasmodium* strains (80–270 nM) and mammalian cell lines (2.1 µM against Vero epithelial cells and 3.1 µM against the MCF-7 cell line). Thus, the bioactivity of palstimolide A is similar to that of other members of this compound class.

In general, this class of polyhydroxy macrolides shows a broad range of biological activity, such as antimalarial, antiparasitic, antifungal, and chemotactic activity [6,8,9,10,11,19,20]. The cytotoxic activity of members of this class is moderate-to-weak (IC_50_s of 0.87 to around 12 µM, with amantelide A being the most cytotoxic; cytotoxic activity for nuiapolide was not reported as a pure compound, however, the crude extract showed no cytotoxic activity at 25 µg/mL).

## 3. Materials and Methods

### 3.1. Chemistry

#### 3.1.1. General Experimental Procedures

Optical rotations were measured on a JASCO P-2000 polarimeter (JASCO Corporation, Tokyo, Japan), UV/Vis data were obtained using a Beckman DU800 spectrophotometer (Beckman Coulter, Brea, CA, USA), and IR spectra were recorded on a Nicolet 100 FT-IR spectrometer (Nicolet, Madison, WI, USA). NMR data were obtained on a Bruker AVANCE III 600 MHz NMR with a 1.7 mm dual tune TCI cryoprobe (Bruker, Billerica, MA, USA) and the 1D TOCSY experiments were performed on a JEOL ECZ 500 NMR spectrometer equipped with a 3 mm inverse probe (H3X) (JEOL Ltd., Tokyo, Japan). Data were recorded in either pyridine-*d5* or methanol-*d4* and adjusted to the residual solvent peak (pyridine-*d5* δ_H_ 7.22, δ_C_ 123.87; methanol-*d4* δ_H_ 3.31, δ_C_ 49.00). Deuterated NMR solvents were purchased from Cambridge Isotope Laboratories (Tewksbury, MA, USA). For high resolution electrospray mass spectrometric analysis (HR–ESI–MS), an Agilent 6530 Accurate Mass QTOF mass spectrometer was used in the positive ion mode (Agilent, Santa Clara, CA, USA). Semi-preparative HPLC was performed on a Thermo Fisher Scientific HPLC system with a Thermo Dionex UltiMate 3000 pump, RS autosampler, RS diode array detector, and automated fraction collector (Thermo Fisher Scientific, Waltham, MA, USA). All solvents were HPLC grade except for water, which was purified by a Millipore Milli-Q system before use.

#### 3.1.2. Extraction and Isolation

A 1 L packed volume of a colorless cyanobacterial collection (voucher specimen available from W.H.G. as collection number RT13 CARTER 10/4) was collected from Palmyra Atoll in the Central Pacific Ocean. Samples were stored in 70% EtOH at −20 °C prior to extraction. Approximately 90 g (dry wt) of cyanobacterial mat was extracted repeatedly with CH_2_Cl_2_/MeOH (2:1) to afford 0.3 g of crude extract (labeled as extract 2238). The material was fractionated by vacuum liquid chromatography (VLC), consisting of TLC-grade (H) silica (Sigma-Aldrich, St. Louis, MO, USA), using a stepwise gradient of increasing polarity, starting with 10% EtOAc in hexanes and finishing with 100% MeOH, to produce nine fractions (B–J). Fractions 2238-H and 2238-I (2238-H eluted with 25% methanol in EtOAc (60.5 mg), 2238-I eluted with 100% methanol (146.7 mg)) were subjected to fractionation via solid phase extraction using a 1.0 g SPE Bond Elut C18 cartridge (Agilent, Santa Clara, CA, USA) with 35% to 100% ACN in H_2_O resulting in five fractions each (H1-H5 and I1-I5). Fractions 2238-H2 and 2238-I2 (eluted with 50% ACN, 2.47 mg and 3.46 mg, respectively) were pooled and subjected to semi-preparative HPLC purification using a linear gradient on a Synergi 5µm Fusion-RP 250 × 10.0 mm column (Phenomenex, Torrance, CA, USA) from (A) H_2_O + 0.1% formic acid to (B) ACN + 0.1% formic acid at a flow rate of 5 mL/min, monitored at 220 nm. The gradient started with 30% B, followed by an increase to 62.5% B in 15 min, and yielded 0.7 mg of compound **1** (RT = 12.5 min).

*Palstimolide A* (**1**). White, amorphous solid; [α]^25^_D_ 13.3 (*c* 0.3, MeOH); UV (MeOH) λ_max_ 213 nm (log ε 3.46), 243 nm (log ε 3.26); IR (KBr) 3393, 2922, 2852, 1579, 1455, 1366, 1249, 1177,1119, 1025 cm^−1^; ^1^H-and ^13^C-NMR, see Table 2; HR-ESI-MS [M + H]^+^ *m/z* 773.6182 (calcd for C_44_H_85_O_10_^+^, 773.6137).

### 3.2. Anti-Leishmanial Testing

#### 3.2.1. Anti-*Leishmania* Assay

*Leishmania donovani* (MHOM/ET/67/HU3) promastigotes were maintained at 28 °C in M199 medium (Gibco, Gaithersburg, MD, USA), supplemented with 10% heat-inactivated fetal bovine serum (FBS, Sigma-Aldrich, St. Louis, MO, USA), 25 mM HEPES, at pH 7.2, and sub-cultured every 4 or 5 days. At 5 days after sub-culture, the parasites were counted and used to infect B10R (murine monocyte cell line), cultivated in DMEM (Gibco, Gaithersburg, MD, USA) supplemented with 10% FBS (Gibco, Gaithersburg, MD, USA) at 37 °C with 5% CO_2_. For the infection, 10,000 cells were mixed with 2 × 10^5^ parasites per well in a 384-well plate in a total volume of 50 µl. For every plate, 16 wells were untreated (negative control) and 16 wells were uninfected (positive control phenotype). Amphotericin B (Sigma-Aldrich #A2942, St. Louis, MO, USA) was used as a reference positive control. Compounds were transferred to plates using an acoustic transfer system (ATS, EDC Biosystems, Fremont, CA, USA) from a stock plate. Compounds were diluted in DMSO and tested at 10 concentrations using a 2-fold serial dilution starting at 10 µM. The maximum DMSO concentration in the assay was 0.1% DMSO. After incubating the plates at 37 °C, 5% CO_2_ for 72 h, the wells were fixed by adding paraformaldehyde for a minimum of 30 min at a final concentration of 2%. After being fixed, the content of the wells was stained with 4′,6-diamidino-2-phenylindole (DAPI, Sigma-Aldrich #10236276001, St. Louis, MO, USA) at 5 µg/mL final concentration. The plate was read using an ImageXpress MicroXL automated microscope (Molecular Devices, San Jose, CA, USA) and 4 images per well were acquired. A custom software was developed for the image analysis to quantify the number of host cells and parasites per well based on size, shape, and intensity of the staining. The antiparasitic activity was calculated based on the relative parasite reduction compared to untreated controls (normalized average = 0% activity) and uninfected wells (normalized average = 100% activity). To calculate the IC_50_ values, GraphPad Prism 6 was used applying a dose-response inhibition nonlinear regression curve fit with variable slope (four parameters). The assay was performed in biological duplicate and the results are presented as an average of the replicates.

#### 3.2.2. Host Cell Toxicity

The host cell toxicity was assessed as part of the anti-*Leishmanial* testing. The average number of host cells in the untreated control wells were counted (=100% viability of the host cells). For every tested well, the percentage of live cells relative to the average of the untreated controls was calculated. Cells were stained and counted according to the procedures described in paragraph 3.2.1. The assay was performed in biological duplicate and the results are presented as an average of the replicates.

### 3.3. Anti-Malarial Testing

#### 3.3.1. Anti-*Plasmodium* Assay

Asexual blood stage *Plasmodium falciparum* Dd2 strain parasites were cultured using standard conditions [21], using RPMI media (Life Technologies, Carlsbad, CA, USA) supplemented with 0.014 mg/mL hypoxanthine (Sigma-Aldrich; prepared fresh), 0.05 mg/mL gentamycin, 38.4 mM HEPES (Sigma-Aldrich), 0.2% sodium bicarbonate (Sigma-Aldrich), 3.4 mM sodium hydroxide (Sigma-Aldrich), 0.05% O+ human serum (Denatured at 56 °C for 40 min; Interstate Blood Bank, Memphis, TN, USA), and 0.0025% albumax (Thermo Fisher Scientific, Waltham, MA, USA). Human O+ whole blood was obtained from the Scripps Research Institute (TSRI) blood bank. Leukocyte-filtered erythrocytes were stored at 50% hematocrit in media as above except without O+ human serum and with 2x albumax concentration at 4 °C. Cultures were monitored every one to two days via Giemsa-stained thin blood smears of parasite infected erythrocytes. Compound potency against asexual blood stage parasites was determined using a SYBR Green I-based fluorescence assay [22]. Asynchronous *P. falciparum* parasites (Dd2 strain) were cultured in standard conditions prior to being assayed. Parasites were initially cultured at 0.6% parasitemia and each compound was tested for anti-parasite potency after 72 h at 37 °C. Compounds were dissolved in DMSO and diluted 1000-fold in the assay resulting in a maximum final DMSO concentration of 0.1% DMSO. Assays were conducted in technical duplicates with three independent biological replicates on a 12-point concentration curve prepared by three-fold dilution from 6.7 μM to 0.11 nM. Artemisinin and chloroquine were simultaneously used as positive controls. IC_50_ values were obtained using normalized SYBR Green I fluorescence intensity, with 6.7 μM artemisinin defined as zero parasite survival, and analyzed via non-linear variable slope four-parameter regression curve-fitting model in Prism 6 (GraphPad Software Inc).

#### 3.3.2. HepG2 Toxicity

The HepG2 toxicity assays were performed as previously reported [23]. Briefly, HepG2-A16-CD81EGFP were cultured at 37 °C in 5% CO_2_ in DMEM (Life Technologies) supplemented with 10% FBS, 0.29 mg/mL glutamine, 100 units/mL of penicillin, and 100 μg/mL streptomycin. A total of 3 × 10^3^ HepG2-A16-CD81EGFP cells in 10 μL of culture medium (DMEM without Phenol Red (Life Technologies), 5% FBS, and 5x Pen Strep Glutamine (Life Technologies)) were seeded in 1536-well plates 24 h prior to treatment. A 50 nL sample of compound was transferred via acoustic transfer system (ATS) (Biosera, Kansas City, MO, USA) into the assay plates and compounds were tested in technical duplicates on a 12-point concentration curve prepared by three-fold dilution from 10.0 μM to 0.15 nM. Compounds were dissolved in DMSO using a maximum DMSO concentration of 0.1% in the assay. Puromycin was used as a positive control for HepG2 cytotoxicity. After 48 h, HepG2-A16-CD81EGFP cell viability was quantified by bioluminescence measurement Envision Multilabel Reader (PerkinElmer, Waltham, MA, USA) using Cell titer Glo (Promega, Madison, WI, USA). IC_50_ values were obtained using the normalized bioluminescence intensity and a non-linear variable slope four-parameter regression curve-fitting model in Prism 6 (GraphPad Software Inc, San Diego, CA, USA).

## 4. Conclusions

In summary, we reported herein the isolation and planar structure elucidation of palstimolide A (**1**), a structurally complex polyhydroxy macrolide. The compound is characterized by a 40-membered macrolactone ring, nine hydroxy group equivalents that form seven 1,5-diol and one 1,7-diol relationships around the ring, a *tert-*butyl moiety, and an α,β-unsaturated carbonyl moiety. Extensive NMR experiments allowed the elucidation of the planar structure despite the small amount isolated (0.7 mg, 0.9 µmol). Palstimolide A shows potent antimalarial activity together with promising preliminary results as an anti-leishmanial agent. This new natural product belongs to a small group of related polyhydroxy macrolides. Members of this natural product class have been isolated from marine Cyanobacteria collected at different tropical regions around the world (e.g., Guam, Hawaii, Panama (Caribbean), and Palmyra Atoll). Likely, these elaborate structures provide an evolutionary advantage for producing cyanobacterial strains; however, this requires further investigation. This structural class appears to be highly attractive for drug discovery efforts because most of these metabolites show a range of biological activities, especially to intracellular parasites.

## Figures and Tables

**Table 1:** NMR spectroscopic data for palstimolide A (**1**) in pyridine-*d*_5_.

#	δ_C_^a^	δ_H_^b^*,* mult (*J* in Hz)	HMBC ^c^	COSY ^d^	TOCSY ^d^
1	167.1	--			
2	117.8	6.01, s	1, 3, 4, 5, 44	4a, Me-44	4a, 4b, 5, Me-44
3	159.9	--			
4a	42.0	3.38, dd (12.5, 3.9)	2, 3, 5, 6, 44	4b, 5	2, 4b, 5, 5-OH, 6a, 6b, 7a, 7b, 8a, 8b
4b		2.92, dd (12.4, 8.8)	2, 3, 5, 6, 44	4a, 5	2, 4b, 5, 5-OH, 6a, 6b, 7a, 7b, 8a, 8b
5	71.1	4.21, b	7	4a, 4b, 6a, 6b	4a, 4b, 5-OH, 6a, 6b, 7a, 7b, 8a, 8b
5-OH	--	5.82, b		5	4a, 4b, 5
6a	39.0	1.78	4, 5, 7, 8	4	4a, 4b, 5
6b		1.74		4	4a, 4b, 5
7a	26.4	1.80	5, 6, 8		
7b		1.58	5, 6, 8, 9		
8a	30.4	1.48, m	7/9, 6/10	7a, 7b/9a, 9b	4a, 4b, 5, 7a, 7b, 9a, 9b, 11
8b		1.42, m	7/9, 6/10	7a, 7b/9a, 9b	4a, 4b, 5, 7a, 7b, 9a, 9b, 11
9a	26.5	1.74	8		
9b		1.58	7, 8, 10, 11		
10	38.4	1.69	8, 11, G2		
11	71.1	3.87	9, 10, G2	10, G2	5, 8a, 8b, G1a, G1b, G2, G3
11-OH	--	5.66, b		11	11
12	G2	G2	G1, G3	G1a, G1b, G3	G1a, G1b, G3
13a	G1	G1a	G2, G3	G2	G2, G3
13b		G1b	G2, G3	(G2)	
14	G2	G2	G1, G3	G1a, G1b, G3	G1a, G1b, G3
15	G3	G3	G1, G2	G2	G1a, G1b, G2, G4
15-OH	--	5.74		G3	
16	G2	G2	G1, G3	G1a, G1b, G3	G1a, G1b, G3
17a	G1	G1a	G2, G3	G2	G2, G3
17b		G1b	G2, G3	(G2)	
18	G2	G2	G1, G3	G1a, G1b, G3	G1a, G1b, G3
19	G3	G3	G1, G2	G2	G1a, G1b, G2, G4
19-OH	--	5.74		G3	
20	G2	G2	G1, G3	G1a, G1b, G3	G1a, G1b, G3
21a	G1	G1a	G2, G3	G2	G2, G3
21b		G1b	G2, G3	(G2)	
22	G2	G2	G1, G3	G1a, G1b, G3	G1a, G1b, G3
23	G3	G3	G1, G2	G2	G1a, G1b, G2, G4
23-OH	--	5.74		G3	
24	G2	G2	G1, G3	G1a, G1b, G3	G1a, G1b, G3
25a	G1	G1a	G2, G3	G2	G2, G3
25b		G1b	G2, G3	(G2)	
26	G2	G2	G1, G3	G1a, G1b, G3	G1a, G1b, G3
27	G3	G3	G1, G2	G2	G1a, G1b, G2, G4
27-OH	--	5.74		G3	
28	G2	G2	G1, G3	G1a, G1b, G3	G1a, G1b, G3
29a	G1	G1a	G2, G3	G2	G2, G3
29b		G1b	G2, G3	(G2)	
30	G2	G2	G1, G3	G1a, G1b, G3	G1a, G1b, G3
31	G3	G3	G1, G2	G2	G1a, G1b, G2, G4
31-OH	--	5.74		G3	
32	G2	G2	G1, G3	G1a, G1b, G3	G1a, G1b, G3
33a	G1	G1a	G2, G3	G2	G2, G3
33b		G1b	G2, G3	(G2)	
34	G2	G2	G1, G3	G1a, G1b, G3	G1a, G1b, G3
35	71.1	3.87	37, G1, G2	36, G2	35-OH, 37a, 39, G1, G2, G3
35-OH	--	5.78		35	
36	38.2	1.78	35, 37, 38, G1, G2		37a
37a	23.5	1.92	38, 39		35, 37b, 38a, 39
37b		1.60	38, 39		
38a	30.2	1.70	37	39	35, 39, 37a
38b		1.64	37, 39, 40	39	35, 39, 37a
39	79.6	5.09, dd (10.3, 1.7)	1, 37, 38, 40, 41-43	38a, 38b	35, 35-OH, 37a, 37b, 38a, 38b
40	34.6	--			
Me-41	26.0	0.94, s	39, 40		
Me-42	26.0	0.94, s	39, 40		
Me-43	26.0	0.94, s	39, 40		
Me-44	26.7	2.09, s	2, 3, 4	2	2

G1: δ_C_ 22.8–22.4, δ_H_ 2.15–2.04/1.89–1.76, G2: δ_C_ 38.7–38.2, δ_H_ 1.82–1.73, G3: δ_C_ 71.2–70.8, δ_H_ 4.00–3.93, G_4_: δ_H_ 5.74; ^a^ Recorded at 150 MHz; referenced to residual pyridine-*d_5_* at δ 123.87 ppm. ^b^ Recorded at 600 MHz; referenced to residual pyridine-*d_5_* at δ 7.22 ppm. ^c^ Carbon showing correlation to indicated proton. ^d^ Proton showing correlation to indicated proton.

**Table 2:** Bioactivity of palstimolide A and bastimolide A (literature data [6]).

Bioactivity Assay	Palstimolide A (95% Confidence Interval)	Bastimolide A
*P. falciparum*	172.5 nM (138.5–214.2 nM)	80–270 nM
*L. donovani*	4670 nM (3067–6273 nM)	3000 nM
HepG2 human liver cell line	5040 nM (4410–5754 nM)	n.d.
B10R murine macrophages (*L. donovani* host cell toxicity)	>10,000 nM	n.d.
Vero epithelial cells	n.d.	2100 nM

## Supplementary Materials

The following are available online: Figure S1: 1H-NMR spectrum of palstimolide A (1) in pyridine-d5, 600 MHz, Figure S2: HSQC spectrum of palstimolide A (1) in pyridine-d5, 600 MHz, Figure S3: Extended 1H-NMR spectrum of palstimolide A (1) in pyridine-d5, 600 MHz, Figure S4: Extended 1H-NMR spectrum of palstimolide A (1) in pyridine-d5, 600 MHz, Figure S5: HMBC spectrum of palstimolide A (1) in pyridine-d5, 600 MHz, Figure S6: LR-HSQMBC spectrum of palstimolide A (1) in pyridine-d5, 600 MHz. Figure S7: COSY spectrum of palstimolide A (1) in pyridine-d5, 600 MHz, Figure S8: TOCSY spectrum of palstimolide A (1) in pyridine-d5, 600 MHz, Figure S9: 1D TOCSY spectrum of palstimolide A (1) in methanol-d4 (500 MHz) with selective irradiation of H5 at 3.78 ppm, Figure S10: 1D TOCSY spectrum of palstimolide A (1) in methanol-d4 (500 MHz) with selective irradiation of H35 at 3.45 ppm, Figure S11: 1D TOCSY spectrum of palstimolide A (1) in methanol-d4 (500 MHz) with selective irradiation of H39 at 4.76 ppm.

## References

[1] Burja A.M., Banaigs B., Abou-Mansour E., Grant Burgess J., Wright P.C. (2001). Marine cyanobacteria—A prolific source of natural products. Tetrahedron.

[2] Tan L.T. (2010). Filamentous tropical marine cyanobacteria: A rich source of natural products for anticancer drug discovery. J. Appl. Phycol..

[3] Niedermeyer T. (2015). Anti-infective Natural Products from Cyanobacteria. Planta Med..

[4] Karpiński T.M. (2019). Marine Macrolides with Antibacterial and/or Antifungal Activity. Mar. Drugs.

[5] Wang M., Zhang J., He S., Yan X. (2017). A Review Study on Macrolides Isolated from Cyanobacteria. Mar. Drugs.

[6] Shao C.-L., Linington R.G., Balunas M.J., Centeno A., Boudreau P., Zhang C., Engene N., Spadafora C., Mutka T.S., Kyle D.E. (2015). Bastimolide A, a Potent Antimalarial Polyhydroxy Macrolide from the Marine Cyanobacterium Okeania hirsuta. J. Org. Chem..

[7] Shao C.-L., Mou X.-F., Cao F., Spadafora C., Glukhov E., Gerwick L., Wang C.-Y., Gerwick W.H. (2018). Bastimolide B, an Antimalarial 24-Membered Marine Macrolide Possessing a tert-Butyl Group. J. Nat. Prod..

[8] Salvador-Reyes L.A., Sneed J., Paul V.J., Luesch H. (2015). Amantelides A and B, Polyhydroxylated Macrolides with Differential Broad-Spectrum Cytotoxicity from a Guamanian Marine Cyanobacterium. J. Nat. Prod..

[9] Mori S., Williams H., Cagle D., Karanovich K., Horgen F., III R., Watanabe C. (2015). Macrolactone Nuiapolide, Isolated from a Hawaiian Marine Cyanobacterium, Exhibits Anti-Chemotactic Activity. Mar. Drugs.

[10] MacMillan J.B., Molinski T.F. (2002). Caylobolide A, a Unique 36-Membered Macrolactone from a Bahamian Lyngbya majuscula. Org. Lett..

[11] Salvador L.A., Paul V.J., Luesch H. (2010). Caylobolide B, a macrolactone from symplostatin 1-producing marine cyanobacteria Phormidium spp. from Florida. J. Nat. Prod..

[12] Andrianasolo E.H., Gross H., Goeger D., Musafija-Girt M., McPhail K., Leal R.M., Mooberry S.L., Gerwick W.H. (2005). Isolation of swinholide A and related glycosylated derivatives from two field collections of marine cyanobacteria. Org. Lett..

[13] Williamson R.T., Boulanger A., Vulpanovici A., Roberts M.A., Gerwick W.H. (2002). Structure and absolute stereochemistry of phormidolide, a new toxic metabolite from the marine cyanobacterium Phormidium sp.. J. Org. Chem..

[14] Pereira A.R., McCue C.F., Gerwick W.H. (2010). Cyanolide A, a glycosidic macrolide with potent Molluscicidal activity from the Papua New Guinea cyanobacterium Lyngbya bouillonii. J. Nat. Prod..

[15] Pereira A.R., Cao Z., Engene N., Soria-Mercado I.E., Murray T.F., Gerwick W.H. (2010). Palmyrolide A, an unusually stabilized neuroactive macrolide from Palmyra Atoll cyanobacteria. Org. Lett..

[16] Wang M., Carver J.J., Phelan V.V., Sanchez L.M., Garg N., Peng Y., Nguyen D.D., Watrous J., Kapono C.A., Luzzatto-Knaan T. (2016). Sharing and community curation of mass spectrometry data with Global Natural Products Social Molecular Networking. Nat. Biotechnol..

[17] Williamson R.T., Buevich A.V., Martin G.E., Parella T. (2014). LR-HSQMBC: A sensitive NMR technique to probe very long-range heteronuclear coupling pathways. J. Org. Chem..

[18] Bax A., Davis D.G. (1985). MLEV-17-based two-dimensional homonuclear magnetization transfer spectroscopy. J. Magn. Reson..

[19] Taori K., Matthew S., Rocca J.R., Paul V.J., Luesch H. (2007). Lyngbyastatins 5-7, potent elastase inhibitors from Floridian marine cyanobacteria, Lyngbya spp.. J. Nat. Prod..

[20] Salvador L.A., Taori K., Biggs J.S., Jakoncic J., Ostrov D.A., Paul V.J., Luesch H. (2013). Potent elastase inhibitors from cyanobacteria: Structural basis and mechanisms mediating cytoprotective and anti-inflammatory effects in bronchial epithelial cells. J. Med. Chem..

[21] Trager W., Jensen J.B. (2005). Human malaria parasites in continuous culture. 1976. J. Parasitol..

[22] Smilkstein M., Sriwilaijaroen N., Kelly J.X., Wilairat P., Riscoe M. (2004). Simple and inexpensive fluorescence-based technique for high-throughput antimalarial drug screening. Antimicrob. Agents Chemother..

[23] Swann J., Corey V., Scherer C.A., Kato N., Comer E., Maetani M., Antonova-Koch Y., Reimer C., Gagaring K., Ibanez M. (2016). High-Throughput Luciferase-Based Assay for the Discovery of Therapeutics That Prevent Malaria. ACS Infect. Dis..

