# Supplementary material for: Palstimolide A: A Complex Polyhydroxy Macrolide with Antiparasitic Activity
Source: Molecules. 2020 Mar 31;25(7):1604. doi: 10.3390/molecules25071604 (PMC7180531; doi:10.3390/molecules25071604)
Supplement: Supplementary file 1 [file molecules-25-01604-s001.pdf]

# Supporting Information

# Palstimolide A, a Complex Polyhydroxy Macrolide with Antiparasitic Activity

Lena Keller[1], Jair L. Siqueira-Neto[2], Julia M. Souza[2,5], Korina Eribez[3], Gregory M. LaMonte[3], Jennifer E. Smith[4], and William H. Gerwick[1,2*]

[1]Center for Marine Biotechnology and Biomedicine, Scripps Institution of Oceanography, University of California San Diego, La Jolla, CA 92037, USA

[2]Skaggs School of Pharmacy and Pharmaceutical Sciences, University of California San Diego, La Jolla, CA 92093, USA

[3]Department of Pediatrics, School of Medicine, University of California San Diego, La Jolla, CA, 92093, USA

[4]Marine Biology Division, Scripps Institution of Oceanography, University of California San Diego, La Jolla, CA 92037, USA

[5]Núcleo de Pesquisas em Ciências Exatas e Tecnológicas, Universidade de Franca, Franca, SP 14404-600, Brazil

***** Correspondence: E-mail: wgerwick@ucsd.edu Tel: +1 (858)-534-0576

## Table of Contents

**Figure S1** $^{1}H$ NMR spectrum of palstimolide A (**1**) in pyridine-$d_5$, 600 MHz.

**Figure S2** HSQC spectrum of palstimolide A (**1**) in pyridine-$d_5$, 600 MHz.

**Figure S3** Extended $^{1}H$ NMR spectrum of palstimolide A (**1**) in pyridine-$d_5$, 600 MHz.

**Figure S4** Extended $^{1}H$ NMR spectrum of palstimolide A (**1**) in pyridine-$d_5$, 600 MHz.

**Figure S5** HMBC spectrum of palstimolide A (**1**) in pyridine-$d_5$, 600 MHz.

**Figure S6** LR-HSQMBC spectrum of palstimolide A (**1**) in pyridine-$d_5$, 600 MHz.

**Figure S7** COSY spectrum of palstimolide A (**1**) in pyridine-$d_5$, 600 MHz.

**Figure S8** TOCSY spectrum of palstimolide A (**1**) in pyridine-$d_5$, 600 MHz.

**Figure S9** 1D TOCSY spectrum of palstimolide A (**1**) in methanol-$d_4$ (500 MHz) with selective irradiation of H5 at 3.78 ppm.

**Figure S10** 1D TOCSY spectrum of palstimolide A (**1**) in methanol-$d_4$ (500 MHz) with selective irradiation of H35 at 3.45 ppm.

**Figure S11** 1D TOCSY spectrum of palstimolide A (**1**) in methanol-$d_4$ (500 MHz) with selective irradiation of H39 at 4.76 ppm.

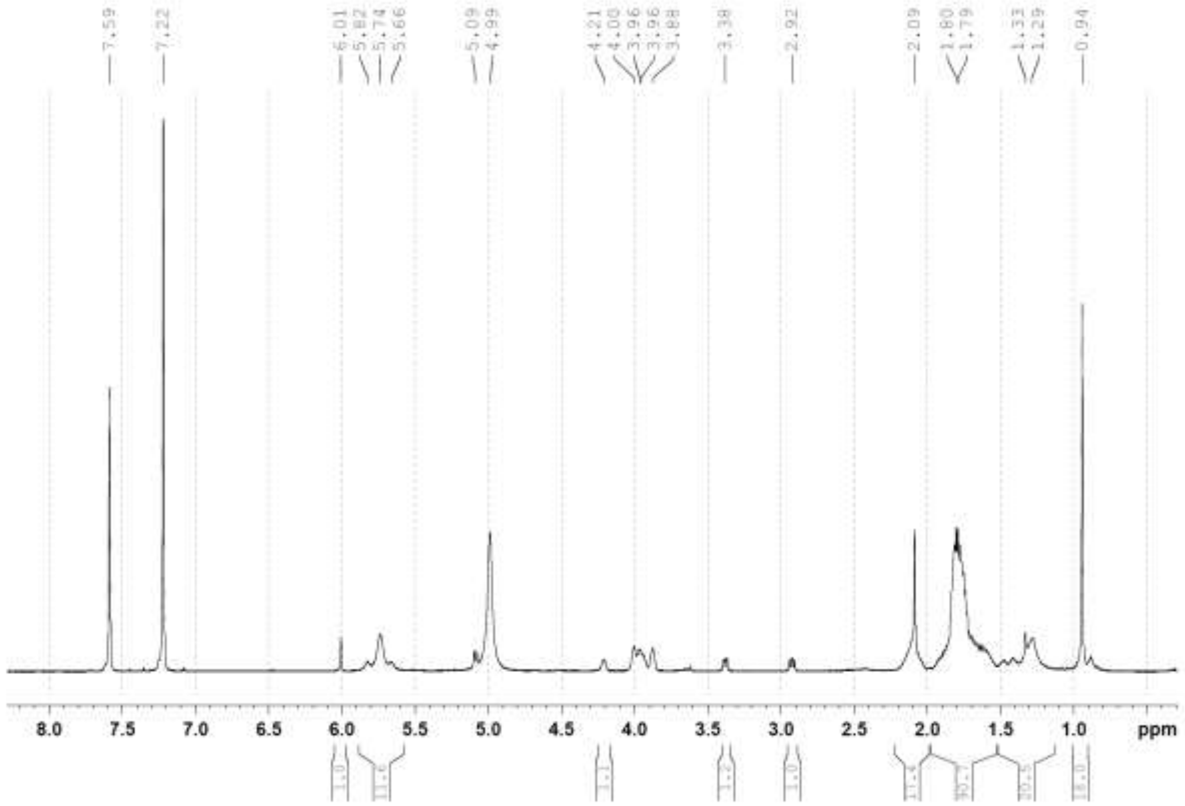

**Figure S1** $^{1}$H NMR spectrum of palstimolide A (**1**) in pyridine-$d_5$, 600 MHz.

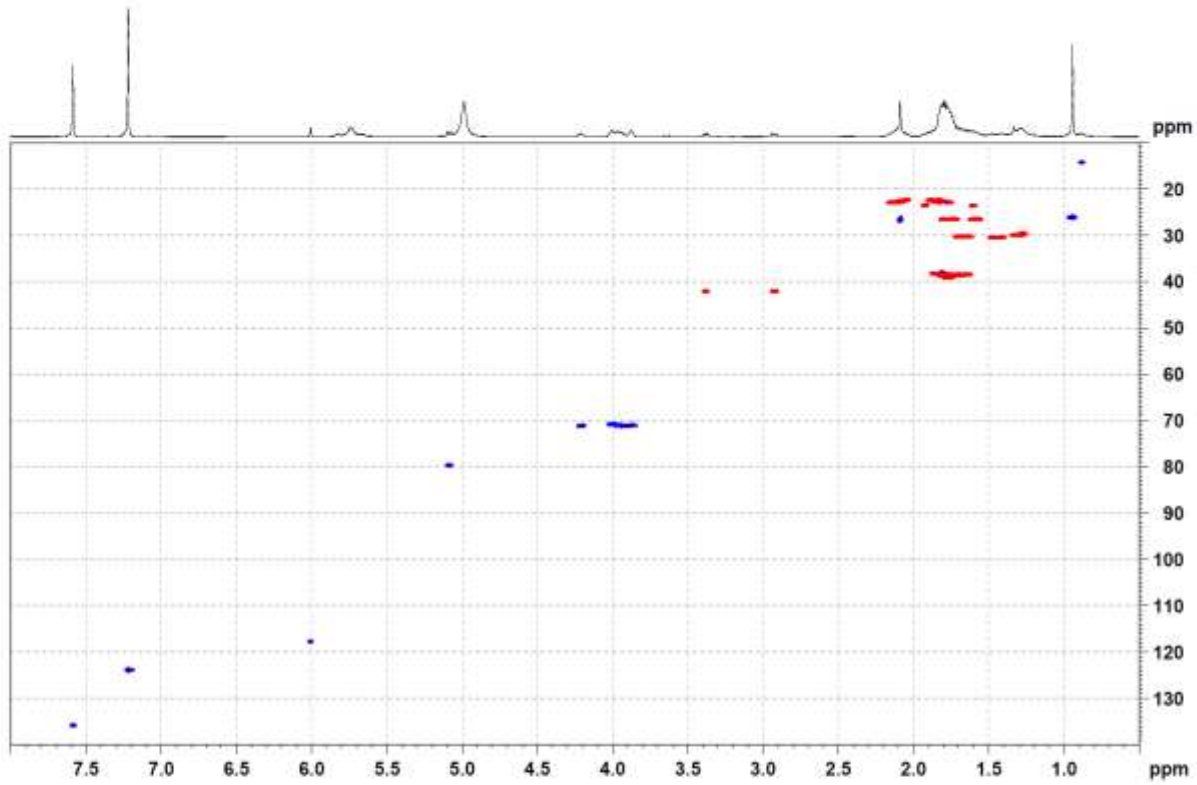


**Figure S2** HSQC spectrum of palstimolide A (**1**) in pyridine-$d_5$, 600 MHz.

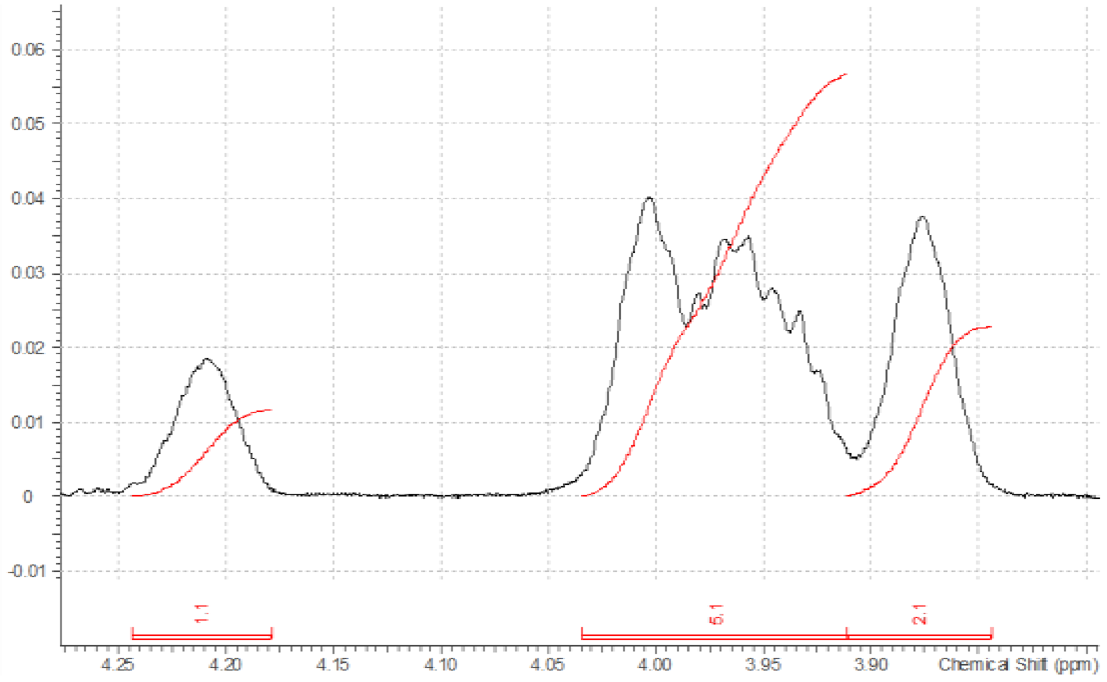


**Figure S3** Extended $^{1}$H NMR spectrum of palstimolide A (**1**) in pyridine-$d_5$, 600 MHz.

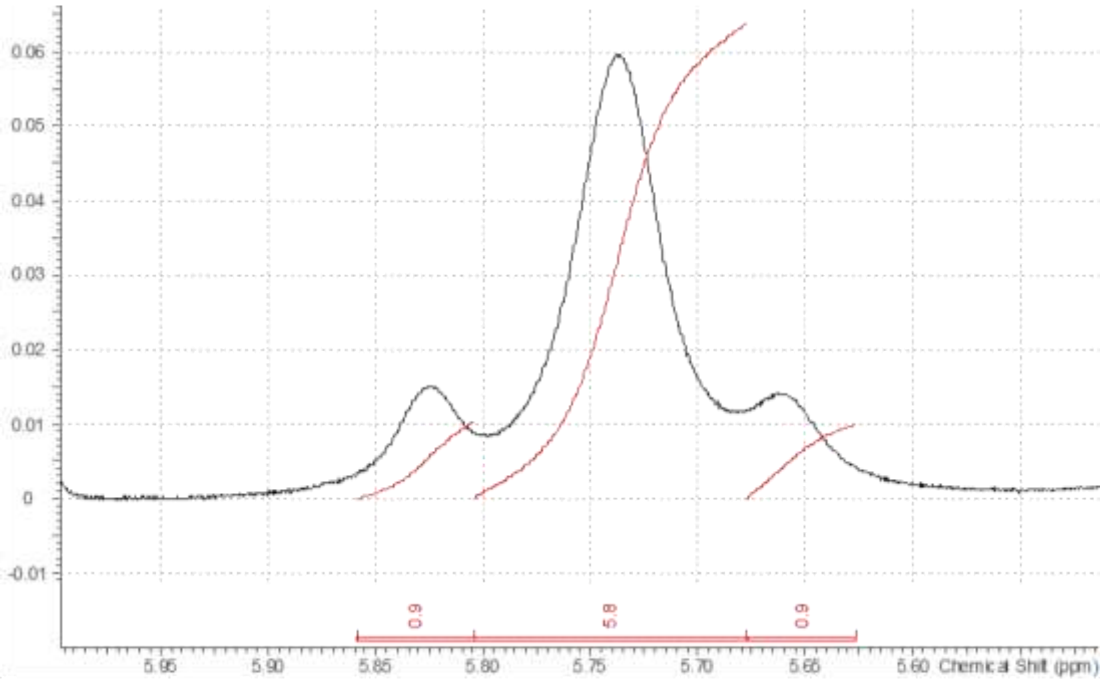


**Figure S4** Extended $^{1}$H NMR spectrum of palstimolide A (**1**) in pyridine-$d_5$, 600 MHz.

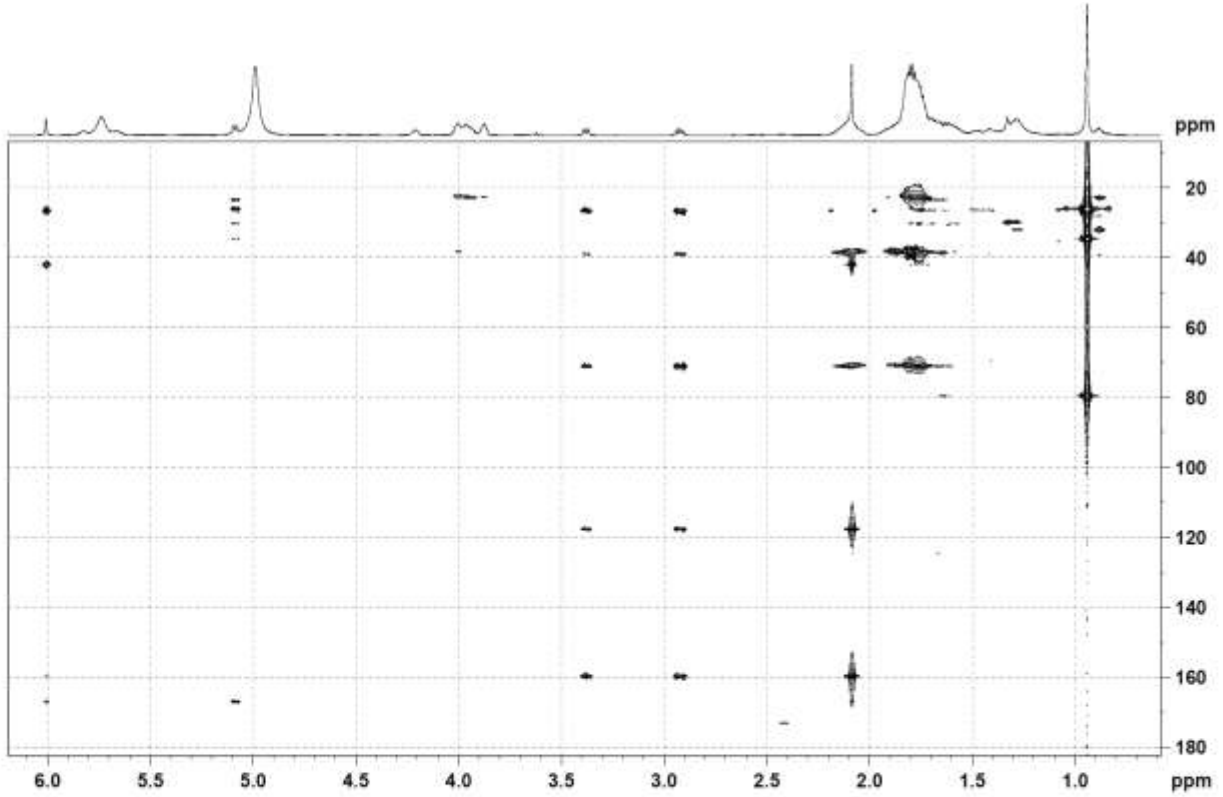

**Figure S5** HMBC spectrum of palstimolide A (**1**) in pyridine-$d_5$, 600 MHz.

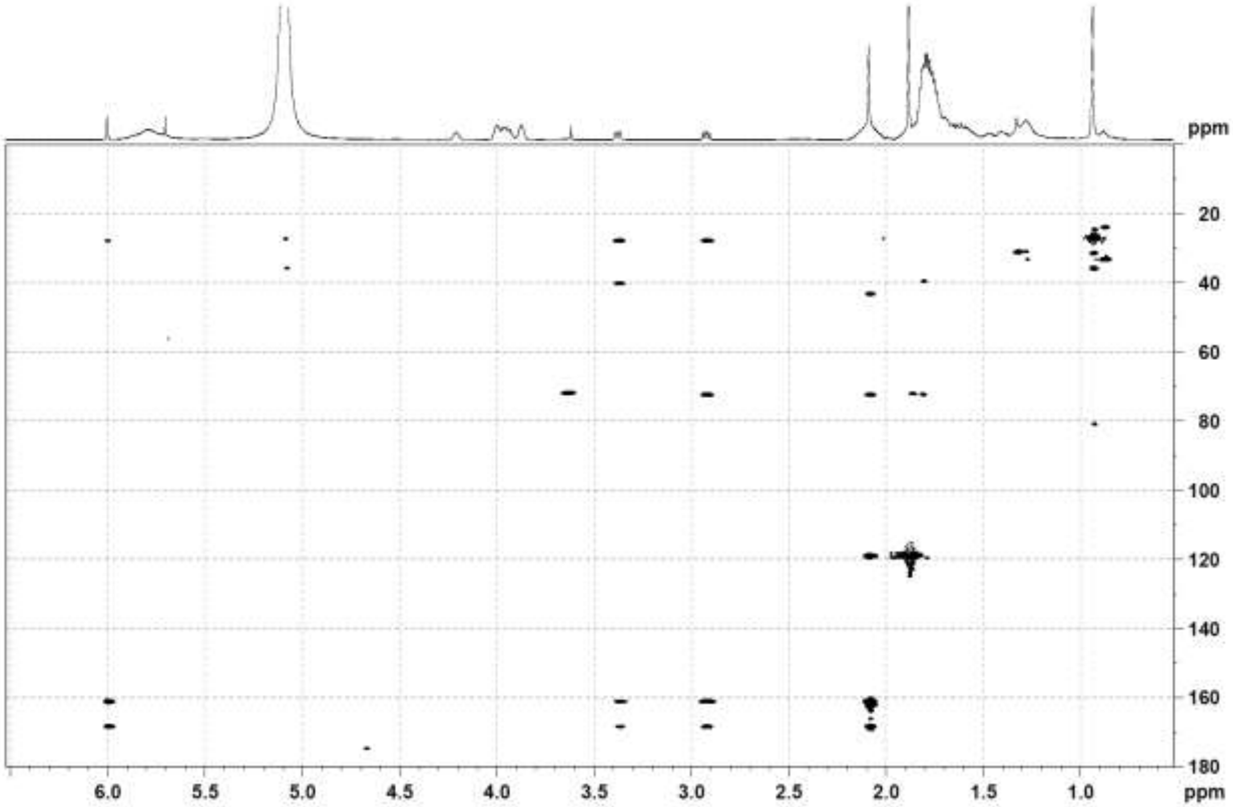


**Figure S6** LR-HSQMBC spectrum of palstimolide A (**1**) in pyridine-$d_5$, 600 MHz.

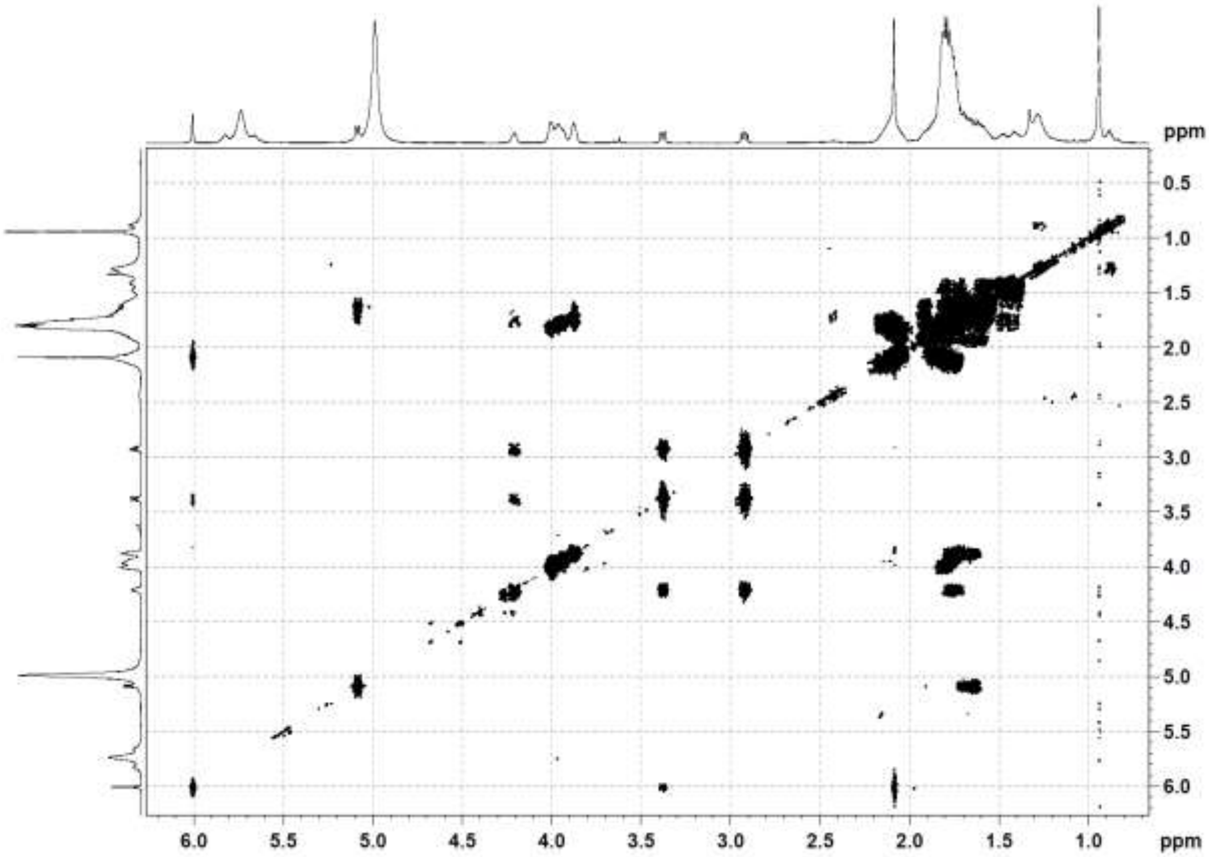

**Figure S7** COSY spectrum of palstimolide A (**1**) in pyridine-$d_5$, 600 MHz.

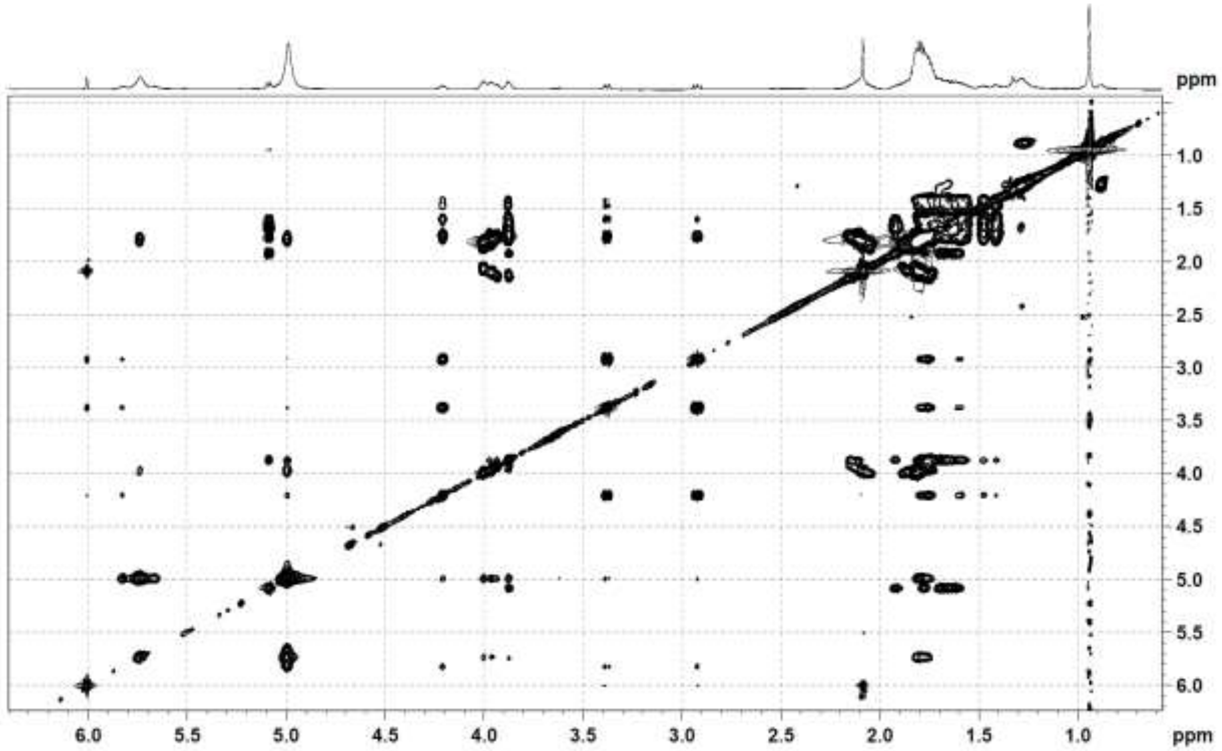


**Figure S8** TOCSY spectrum of palstimolide A (**1**) in pyridine-$d_5$, 600 MHz.

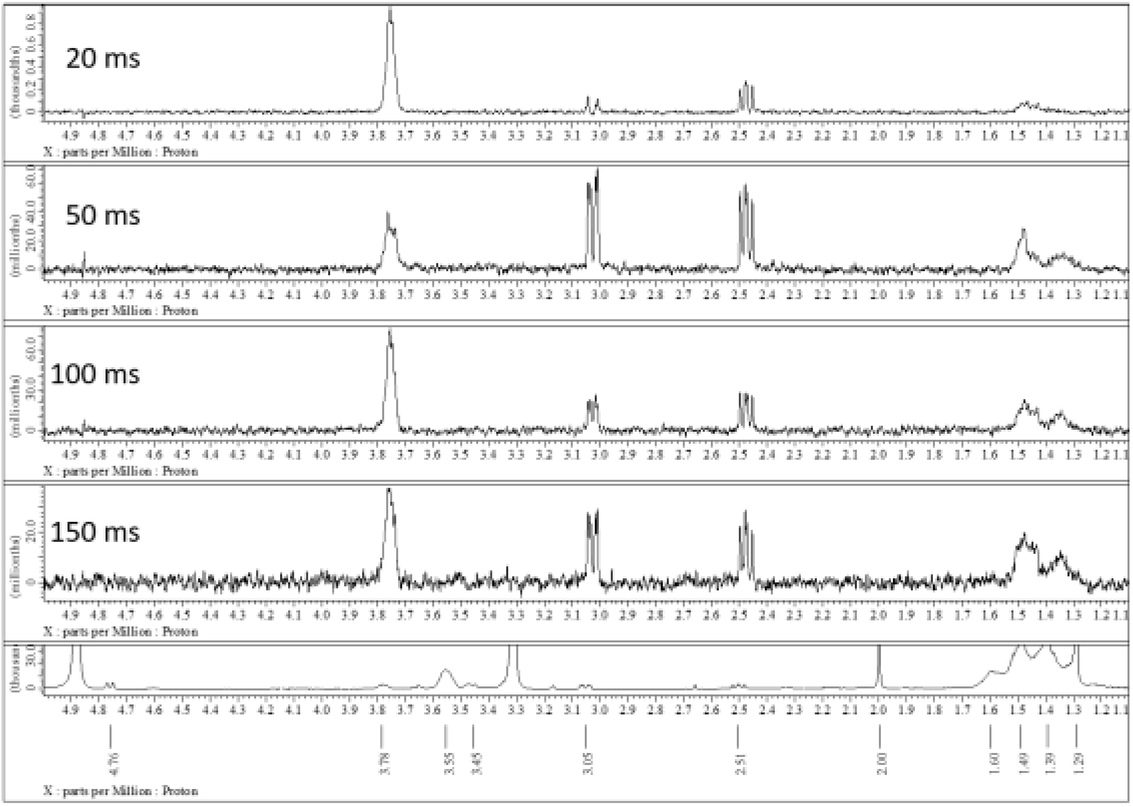


**Figure S9** 1D TOCSY spectrum of palstimolide A (**1**) in methanol-$d_4$ (500 MHz) with selective irradiation of H5 at 3.78 ppm.

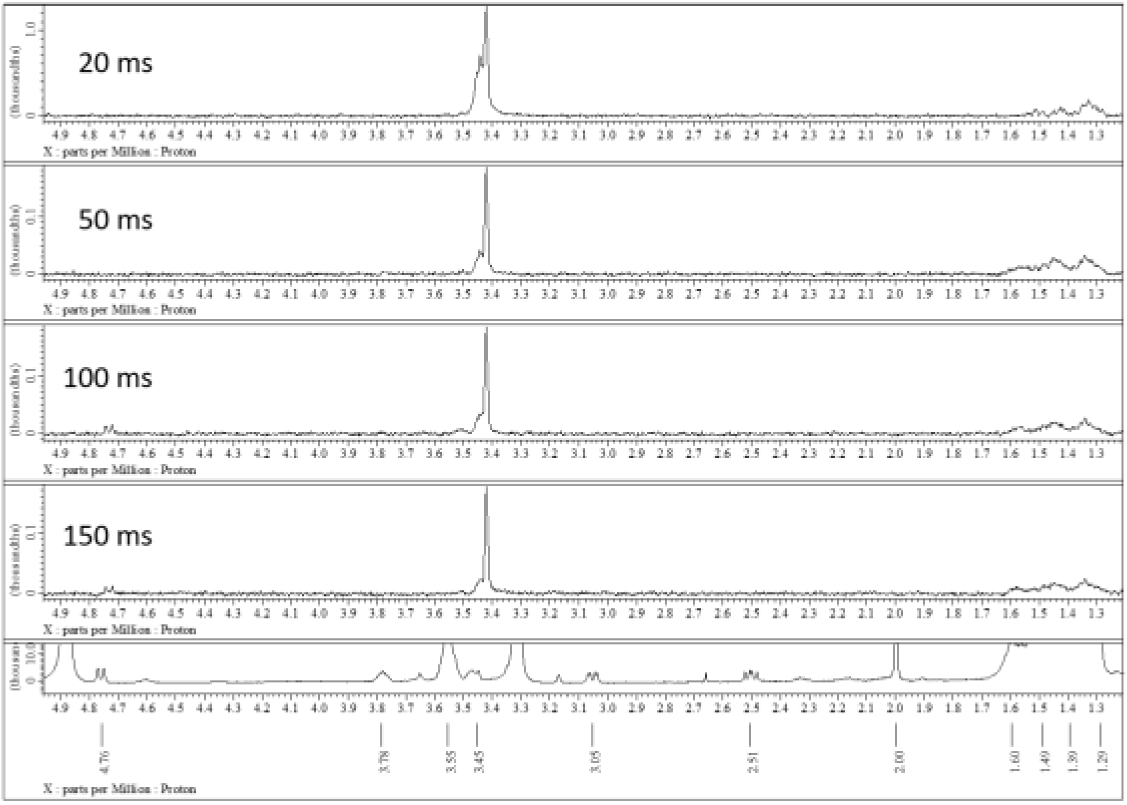


**Figure S10** 1D TOCSY spectrum of palstimolide A (**1**) in methanol-$d_4$ (500 MHz) with selective irradiation of H35 at 3.45 ppm.

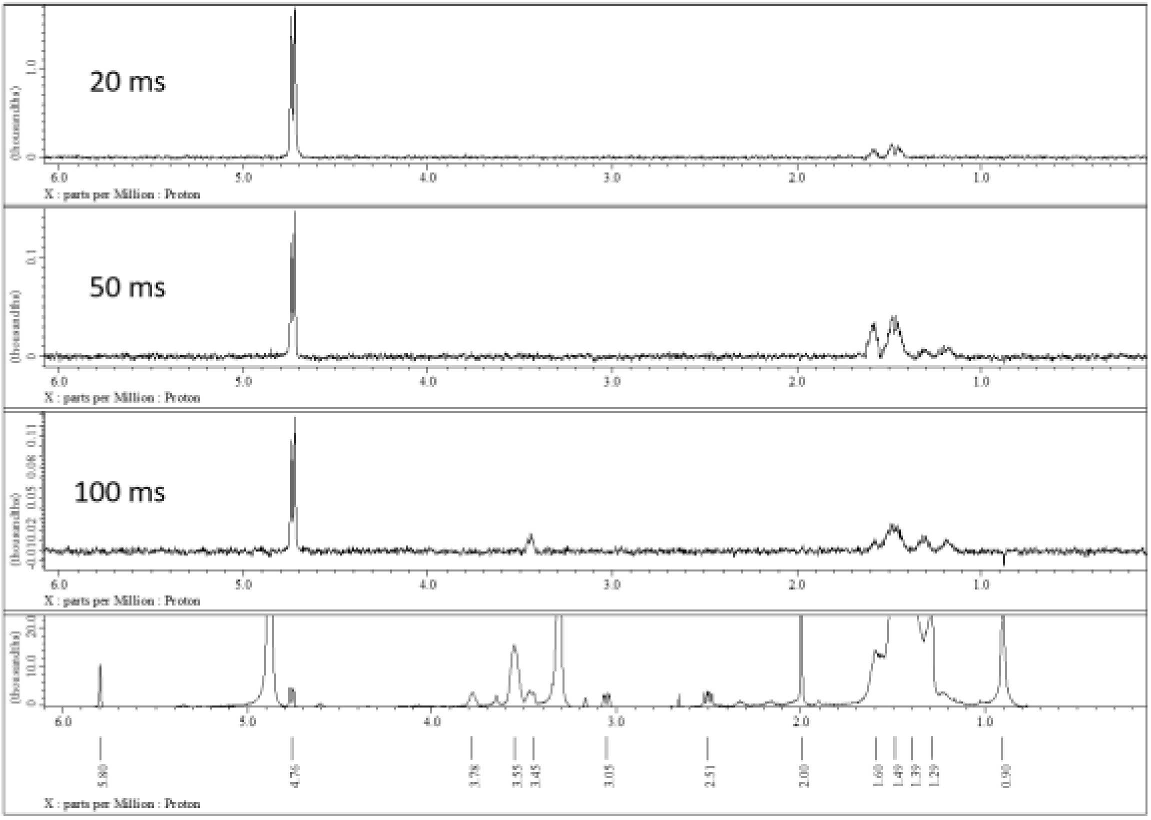


**Figure S11** 1D TOCSY spectrum of palstimolide A (**1**) in methanol-$d_4$ (500 MHz) with selective irradiation of H39 at 4.76 ppm.
